# Pre-Treatment with Bromelain Prevents Intestinal Dysbiosis in Pigs with Post-Weaning Diarrhea, without Increasing Antimicrobial Resistance in *Escherichia coli*

**DOI:** 10.3390/ani13203229

**Published:** 2023-10-16

**Authors:** Alison Collins, Bethany Bowring

**Affiliations:** 1 New South Wales Department of Primary Industries, Elizabeth Macarthur Agricultural Institute, Menangle, NSW 2568, Australia; 2 Centre for Infectious Diseases and Microbiology, the Westmead Institute for Medical Research, Sydney, NSW 2145, Australia; bethany.bowring@sydney.edu.au

**Keywords:** antimicrobial resistance, bromelain, dysbiosis, enterotoxigenic *Escherichia coli*, microbiome, neomycin, zinc, post-weaning diarrhea

## Abstract

**Simple Summary:**

Antimicrobials are commonly used to prevent diarrhea and poor growth in the first two weeks after pigs are weaned, but they also produce side effects including the emergence of antimicrobial resistance and the destruction of protective bacteria that can exclude pathogens. Bromelain, an enzyme extract from plants, blocks attachment of the pathogen *Escherichia coli* to host cells, so it was evaluated against neomycin and zinc oxide for the prevention of diarrhea, reduction in pathogenic *E. coli*, disruption of the gut bacterial population and the emergence of antimicrobial resistance. All treatments prevented diarrhea in weaner pigs, but only bromelain was able to maintain the stability of the gut bacterial population and prevent the emergence of antimicrobial resistance in gut bacteria. On-farm studies have shown that bromelain can prevent post-weaning diarrhea, but this is the first study to show bromelain’s ability to stabilize gut bacterial populations in the critical post-weaning period. Bromelain treatment, in combination with probiotics, good hygiene and low-protein diets, may reduce the reliance on antibiotics and heavy metals for preventing post-weaning diarrhea, which will ultimately benefit both pig and human health.

**Abstract:**

Pigs are especially vulnerable to intestinal pathogens and dysbiosis in the first two weeks after weaning. Infection with enterotoxigenic strains of *Escherichia coli* (ETEC) in combination with poor nutrition and hygiene can lead to diarrhea, poor growth and increased mortality. While neomycin and zinc oxide can prevent post-weaning diarrhea (PWD), their broad-spectrum activity also kills commensal microbiota and can lead to the emergence of heavy metal and antimicrobial resistance. Bromelain prevents attachment of F4 ETEC to intestinal enterocytes by cleaving the host receptor. In controlled environmental facilities, weaned pigs treated with either therapeutic levels of neomycin sulfate, zinc oxide, bromelain or non-treated were monitored for diarrhea, weight gain, feed intake, feed efficiency, excretion of F4 ETEC, changes to their intestinal microbiomes and antimicrobial resistance in *E. coli*. The treatment effects were evaluated at weaning, during two weeks of treatment and for three weeks after treatments ceased. Minimal clinical signs of PWD were observed, except in zinc-treated pigs post treatment. Intestinal dysbiosis was observed in response to diarrhea and in pigs treated with both neomycin and zinc. Antimicrobial resistance increased in commensal *E. coli* isolated from neomycin- and zinc-treated pigs. In contrast, bromelain controlled PWD and prevented intestinal dysbiosis without inducing antimicrobial resistance.

## 1. Introduction

Pigs are especially vulnerable to intestinal pathogens in the first two weeks after weaning due to the loss of maternally derived immunoglobulins, the mixing of litters exposing piglets to new bacterial populations and disturbances in the commensal and pathogenic microflora caused by abrupt dietary changes [1,2,3]. These changes at weaning can induce intestinal dysbiosis, characterized by a reduction in overall bacterial diversity or the loss of beneficial microbes [4]. Commensal bacteria, including Lactobacilli, play an important role in preventing the colonization of pathogens through competitive exclusion and excretion of bacteriocins capable of bacterial lysis [5]. Enterotoxigenic *E. coli* (ETEC), previously inhibited by commensal bacteria or inactivated by maternal antibodies, can now attach to the host epithelium by fimbriae (type F4 in weaner pigs) and inject toxins that induce the secretion of water into the intestinal lumen, causing diarrhea [6]. The integrity of the intestinal barrier is compromised during ETEC infections [7], potentially increasing the translocation of bacteria and their toxins through the epithelial barrier. Both the damage to the intestinal barrier and dysbiosis of the intestinal microbiome can lead to chronic inflammation and metabolic dysfunction in the piglet [8,9]. Mortality due to post-weaning diarrhea (PWD) can be as high as 25% [10] but usually ranges between 1.5 and 2% [11].

Antimicrobials are commonly used to treat or prevent post-weaning diarrhea (PWD) in pigs, although there is evidence that bacteria can develop resistance to antimicrobials [12], and broad-spectrum antibiotics can kill commensal bacteria and cause dysbiosis of the intestinal microbiome [13,14]. Neomycin sulfate is a broad-spectrum aminoglycoside that has bactericidal activity against porcine ETEC [15] and can reach therapeutic concentrations in the intestinal lumen [16]. Although resistance to neomycin in *E. coli* isolated from PWD has been reported, surveys in Australia indicate that resistance to neomycin is less common than resistance to other commonly used antibiotics including streptomycin, spectinomycin, ampicillin and trimethoprim-sulphamethoxazole [17].

Therapeutic concentrations of zinc oxide, between 2400 and 3000 ppm, have also been routinely used in the first two weeks post-weaning to reduce the incidence and severity of diarrhea and improve growth [18]. Zinc is reported to block pathogen and toxin invasion by preventing increased permeability of tight junctions between epithelial cells [19]. However, the high concentration of zinc in manure has led to concerns about environmental contamination and the emergence of methicillin-resistant *Staphylococcus aureus* (MRSA) in pigs fed high zinc levels [12].

Bromelain is a proteolytic enzyme extracted from the stem of *Ananas comosus* reported to have phytomedical properties, capable of protecting pigs from PWD and the associated production losses under commercial conditions [20]. Bromelain causes proteolytic cleavage of F4 ETEC receptors on host enterocytes and therefore prevents attachment of F4 ETEC to the piglet small intestine [21]. Bromelain also inhibits intestinal fluid secretion caused by ETEC toxins by mediating adenosine 3:5 -cyclic monophosphatase, guanosine 3:5 -cyclic monophosphatase and calcium-dependent signaling cascades [22]. Importantly, as bromelain acts on the host ETEC receptors and not on bacterial structure or function, it is not expected to have any antimicrobial activity. 

The intestinal microbiome plays a significant role in host digestion, immune development and homeostasis of the intestine. Pigs usually recover from PWD when their immune systems mature and their microbiomes stabilize. Diarrhea and treatments that cause dysbiosis of the intestinal microbiome limit the host’s ability to protect itself from disease and recover after damage. This study compares the impact of bromelain, neomycin sulfate and zinc oxide on PWD, pig growth, intestinal permeability and the stability of the intestinal microbiome in the six weeks after weaning. It is hypothesized that all treatments will prevent PWD, but intestinal dysbiosis and increased antimicrobial resistance will only be prevented in pigs treated with bromelain.

## 2. Materials and Methods

### 2.1. Animals, Housing and Experimental Design

**Figure 1:**
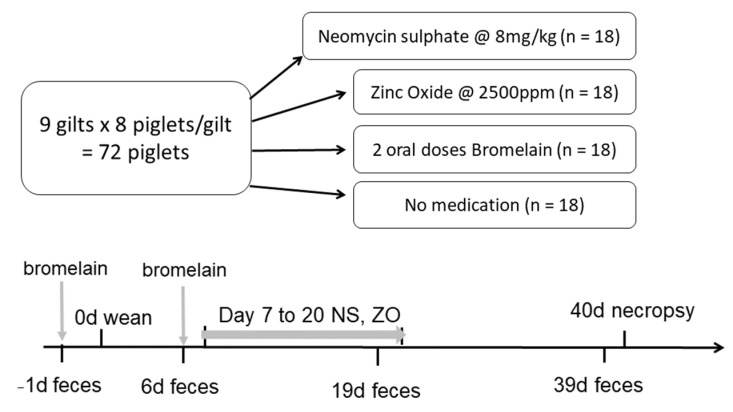
Schematic of randomization of piglets and timeline of experimental design for pigs treated with bromelain, neomycin sulfate (NS) or zinc oxide (ZO) or not medicated.

A total of 72 Large White Landrace piglets were sourced from a high-health-status commercial piggery, where dams were fed non-medicated lactation diets. Sows’ teats were sprayed with an autogenous Lactobacilli species in the week before weaning. Eight medium-weight piglets were selected from each of nine litters, and two piglets per litter (n = 18) were allocated into each of four treatment groups at weaning, balancing for litter and weaning weight. Piglets allocated to the bromelain treatment were dosed orally with 4 mL of proprietary bromelain (50,000 CDU/mL) the day before weaning (day −1) and again at day 6 post weaning. All piglets were transported to the research facility at weaning (day 0) and given one week to acclimatize in temperature-controlled rooms set to 24 °C ± 2 °C, with ad lib access to a non-medicated low-protein creep feed (175 g crude protein/kg). The study was conducted according to the Australian Code for the Care and Use of Animals for Scientific Purposes, and animal ethics approval was granted by the Animal Ethics Committee of Elizabeth Macarthur Agricultural Institute (EMAI, M16/02). Each treatment was housed in a separate room with strict biosecurity between rooms. Within treatments, pigs were randomly allocated into six pens of three pigs per pen. Neomycin sulfate (8 mg/kg body weight) and zinc oxide (2500 ppm) treatments commenced in a low-protein weaner feed from day 7 to day 20 post-weaning (Figure 1). Between days 20 and 40 post weaning, all pigs were maintained on a commercial non-medicated weaner feed. Pigs were euthanized at 40 days post weaning, and intestinal tissue was collected for microbiome analysis and genetic testing for the MUC4 genetic marker associated with F4 ETEC susceptibility/resistance.

### 2.2. Production Measures and Analysis

Feed intake per pen and weight gains per pig were recorded weekly. Feed efficiency was calculated as the ratio of weight gain to feed intake. The effect of treatment on production was analyzed using an unbalanced ANOVA with replicate (pen), gender, litter and their interactions as blocking effects (GenStat, 18th edition). Repeated-measures ANOVA was used to test the effect of time on the same production parameters. For both analyses, starting weight was used as a covariate when analyzing pig weight, weight gain, feed intake and feed efficiency. Differences were determined using Fisher’s Least Significant Difference test. Statistical significance was accepted at *p* < 0.05.

### 2.3. Fecal Sampling and Analysis

Fecal consistency was evaluated daily on a score of 1 to 4 (1 = normal consistency; 2 = semisolid without blood; 3 = watery with no blood or dark feces; 4 = blood-tinged feces), and the number of days with a diarrhea score ≥ 2 was tallied. The duration of diarrhea was calculated as the number of consecutive days in each period where pigs had a fecal consistency ≥ 2. Individual feces were collected from each sow and piglet pre-weaning (day −1) and from weaned pigs before in-feed treatments started (day 6), the day before treatments ceased (day 19) and 20 days after treatments ceased (day 39). Feces were stored at −20 °C for less than one week before nucleic acids were extracted for microbiome analysis.

Aliquots of fecal supernatants from days 19 (during medication) and 39 (after medication) were clarified in Carrez solutions I and II, and precipitated proteins were removed by centrifugation at 10,000× *g* for 5 min prior to quantifying the concentration of D- and L-lactate in samples (D- and L-Lactate ELISA kit, Megazyme International, Wicklow, Ireland). Concentrations of lactate were expressed as millimolar concentrations per dry weight of feces to minimize the effect of higher water content in scouring piglet feces. Lactate concentrations were compared between treatments using an unbalanced ANOVA, and the effect of time on lactate concentrations was measured with a repeated-measures ANOVA. Fecal dry weight was determined by measuring the weight difference after freeze-drying.

### 2.4. Nucleic Acid Extraction and Quantitative PCR

Approximately 0.1–0.2 g of each fecal sample was used for DNA extraction with the MagMAX Pathogen RNA/DNA kit (Applied Biosystems, Waltham, MA, USA) on a magnetic particle processor (Biosprint 96, Qiagen, Venlo, Netherlands). The numbers of F4 *E. coli*, total *E. coli*, *Enterobacteriaceae* and total bacteria were quantified by real-time PCR (qPCR) as previously described [23,24] using standards constructed from known numbers of the appropriate bacterial culture. TaqMan probes were synthesized with 5-carboxyfluorescein (FAM) on the 5′ end and Black Hole Quencher (BHQ-1) on the 3′ end. Each microbial quantitative PCR (qPCR) reaction contained 1× reaction buffer, 1U Taq polymerase (AgPath-ID RT-PCR kit, Applied Biosystems), 5 pmol of each primer, 1 pmol of probe and 5 μL of DNA (diluted 1/10 or 1/100 for the total bacteria qPCR) in a 25 μL volume.

All PCR reactions were prepared using the EpMotion 5075 liquid-handling robot, (Eppendorf, Enfield, CT, USA) and targets were amplified with the ViiA7 384-well PCR machine (Applied Biosystems, Waltham, MA, USA). The initial denaturation was performed at 95 °C for 10 mins, followed by 40 cycles of 95 °C for 15 s and annealing for 40 s. Annealing was performed at 63 °C for *Enterobacteriaceae* and total *E. coli* but was modified to 58 °C and 60 °C for total bacteria and F4 *E. coli*, respectively. Standards were assayed in duplicate, and linear regressions of standard curves were confirmed to have an R^2^ > 0.98 and a PCR efficiency between 90% and 110%. Bacterial numbers were expressed as a ratio of the total bacteria number in the same sample. Bacterial ratios were log10-transformed to normalize the data. The effect of treatment on different bacterial ratios was analyzed using an unbalanced ANOVA. Gender and litter were included as blocking effects, and treatment structure was included as pens within treatment groups. Fisher’s least significant difference (LSD) tests were used to compare the means of different treatment groups.

### 2.5. Next-Generation Sequencing and Microbiome Analysis

The concentration of DNA extracted from each sample was quantified with the Qubit dsDNA BR assay on the Qubit fluorometer (Invitrogen Life Technologies, Waltham, MA, USA), and 75 ng of each extract was submitted for 16S rRNA V4 (515f-806r) amplicon library preparation and sequencing (Ramaciotti Centre for Genomics, UNSW). Sequencing was conducted on the Illumina MiSeqV2, with 2 × 250 bp paired-end sequences. Paired-end reads were analyzed in the Qiagen CLC Genomics workbench v21. Sequencing adapters were trimmed, and samples were filtered to remove chimeras, low-abundance reads and short reads. Overlapping forward and reverse paired reads were merged to produce one high-quality read. 

Operational Taxonomic Unit (OTU) clustering was performed according to the manufacturer’s instructions (OTU Clustering Step by Step, Qiagen 2019) using the reference database provided (16S_97_otus_GG.clc) and filtering out low-abundance OTUs (less than 10). The metadata were aggregated with the OTU table produced from clustering analysis. A phylogenetic tree of all OTUs was constructed using a maximum likelihood analysis based on multiple sequence alignment of the OTUs generated by MUSCLE. Alpha diversity analysis used this phylogenetic tree to provide an estimate of the diversity of bacteria within a sample. Rarefaction analysis set the minimum and maximum depths to sample at 1 and 5000, with 20 depths to be sampled and 100 replicates at each depth. Rarefaction plots were checked for plateauing of the phylogenetic diversity in all samples, to indicate good coverage of bacterial sequences. Significant differences in the mean phylogenetic diversity between treatments were determined by an unbalanced ANOVA using the nonparametric Kruskal–Wallis test.

Beta diversity was measured by UniFrac, an analysis tool used to determine whether bacterial communities were significantly different between samples, displaying the relatedness with principal coordinates analysis (PCoA). Significant dissimilarities in the composition of microbial communities between treatments were analyzed by the Bray–Curtis statistic using permutational multivariate analysis of variance (PERMANOVA). Linear discriminate analysis (LDA) on effect size was performed to determine the combination of OTUs (bacterial groups) that most likely explain the microbiome differences between treatments using a score of greater than Log_10_2 for statistical significance (*p* ≤ 0.05) [25]. 

### 2.6. Phenotypic Antimicrobial Resistance

*Escherichia coli* were cultured from fresh feces on the selective tryptone bile X-glucuronide (TBX) agar. Blue *E. coli* colonies were subcultured onto sheep blood agar to test for hemolysis, and then the susceptibility of four *E. coli* isolates from each pig at each time point was tested phenotypically against 7 commonly used antimicrobials (amoxicillin, apramycin, lincospectin, neomycin, sulphamethoxazole/trimethoprim, and tetracycline) according to the CDS disc diffusion method [26]. As isolates were either resistant or susceptible, a binomial distribution was assumed. Significant differences between treatments in the proportion of resistant *E. coli* for each antibiotic were determined by logistic regression, with individual treatments compared by least significant differences (LSD). Multidrug-resistant *E. coli* proportions were analyzed in the same way. Changes in the proportion of resistant *E. coli* over time were calculated, and significant effects of treatment on the proportion of resistant *E. coli* were analyzed with a Kruskal–Wallis one-way ANOVA.

## 3. Results

### 3.1. Diarrhea Scores and Treatments

Prior to the in-feed treatments commencing (days 0 to 6), pigs selected for the zinc oxide and control groups had short-term diarrhea that was effectively controlled with a single dose of electrolytes (Vytrate™, Jurox, Rutherford, NSW, Australia). Between 7 and 20 days post weaning, diarrhea was observed in all treatments, requiring electrolytes on more than two consecutive days for control, bromelain and zinc-treated pigs (Table 1). Within 5 to 7 days of removing neomycin sulfate and zinc oxide from diets (days 21 to 39), approximately 25% of pigs in these groups developed diarrhea that required electrolytes on three consecutive days. Percent fecal water content was higher in zinc-treated pigs relative to neomycin and bromelain pigs after treatments ceased (Table 2). Fecal water content was also lower in the digesta of bromelain-treated pigs at necropsy. Time had a significant effect on the percent water in feces, with the percent fecal water increasing between 6 and 19 days, then decreasing between 19 and 39 days, after each treatment ceased. The percent water content in pig feces at day 6 was correlated with the diarrhea score between days 0 and 6 (r = 0.56). Likewise, the fecal water content at day 19 was correlated with the diarrhea score between days 7 and 20 (r = 0.52). However, the percent fecal water at 39 days did not correlate highly with the diarrhea score of pigs between days 21 and 39 (r = 0.11). There were no significant differences in the mean diarrhea score (*p* = 0.384), duration of diarrhea (*p* = 0.082), or number of Vytrate doses between treatments (*p* = 0.610) over the whole trial period (Table 1 and Table 2). The number of Vytrate treatments was also not affected by time (repeated-measures ANOVA not shown).

The double-recessive F4 ETEC-resistant genotype (MUC4 marker) varied widely between litters (14% to 100%), but random allocation of piglets to treatments reduced variation in F4 ETEC susceptibility between treatments, with 61% of controls, 67% of bromelain-, 50% of neomycin- and 71% of zinc-treated piglets carrying the resistant genotype. The F4 ETEC genotype had no effect on the diarrhea score or number of Vytrate doses, except in the first week before treatment (days 0 to 7 post weaning) when counterintuitively resistant pigs required more Vytrate doses (*p* = 0.073) due to increased diarrhea severity (*p* = 0.003) than F4 ETEC-susceptible pigs. Genetic resistance to the F4 ETEC receptor (MUC4) had no effect on the relative abundance of F4 ETEC detected in mucosal scrapings at necropsy nor in feces at 6, 7, 19 or 39 days post weaning across all treatments (*p* > 0.05). 

Numbers of F4 ETEC detected in feces were predominantly below 10^4^ ETEC per gram of feces but were detected at up to 5 × 10^6^ in a small proportion of pigs at day 6 post weaning. Numbers of F4 ETEC and total bacteria in mucosal scrapings were significantly lower than in feces, but the relative abundance of F4 ETEC was similar between fecal and mucosal samples. Surprisingly, the numbers of F4 ETEC in feces were significantly increased in MUC4 genetically resistant zinc-treated pigs compared to their susceptible cohorts after zinc treatment ceased (day 39) (*p* = 0.034). 

### 3.2. Production Performance

Weaning weights were not significantly different between treatments (*p* > 0.05); however, weight gain was significantly increased in neomycin-treated pigs compared to bromelain-treated pigs during the 32-day experimental period (Table 3). Weight gain was affected by time and interactions between time and treatment, with weekly weight gains increasing to a peak between 21 and 28 days, then decreasing between 28 and 35 days. Early weekly weight gain was also affected by litter but not by gender or replicate (pen). Although feed intake was significantly increased in neomycin-treated relative to bromelain-treated pigs, feed efficiency (feed:gain) was similar between treatments (*p* > 0.05). Feed intake was also affected by litter, gender, pen, time, interactions between pen and treatment and interactions between time and treatment. Feed intake increased significantly between week 1 and week 2 and again between weeks 3 and 4. Feed efficiency was not affected by any other factors in the experimental design. Two neomycin-treated pigs died on days 19 and 39 due to a *Streptococcus suis* infection and chronic pericarditis, respectively. However, mortality was not significantly different between neomycin (11%) and other treatments (0%).

### 3.3. Microbiome Analysis

**Figure 2:**
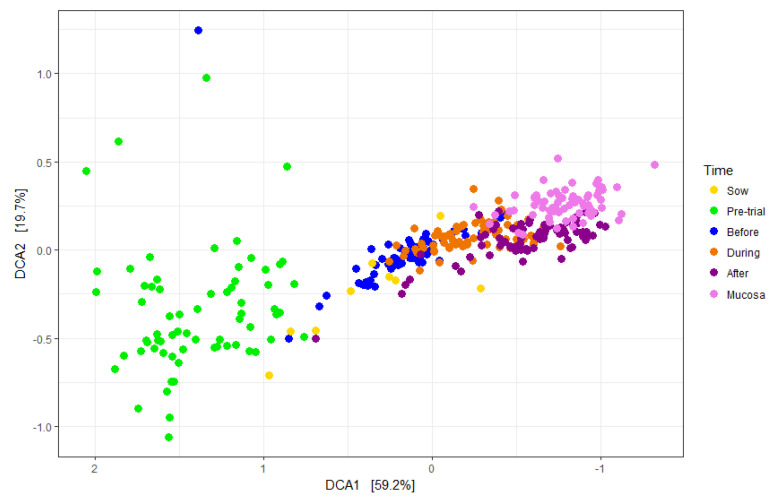
Detrended canonical analysis showing the beta diversity of microbiomes between individuals of the same age and between different-aged pigs. Distances between data points are linear in both the x and y directions.

The most common phyla found in weaner pig feces were Firmicutes (42.06%), Bacteroidetes (40.54%) and Proteobacteria (6.60%). *Bacteroidetes* consisted predominantly of *Prevotellaceae*; *Firmicutes* consisted predominantly of *Clostridiales*, encompassing *Veillonellaceae*, *Ruminococcaceae and Lachnospiraceae*, but also included *Lactobacillaceae*. Age was a significant factor in fecal microbiomes, with the microbiomes of pre-weaned pigs clustering most closely to their dams and separately from the microbiomes of post-weaned pigs (Figure 2). Repeated-measures ANOVA demonstrated a significant increase in the relative proportions of F4 ETEC over time from a peak at day −1 (−3.60) to a low at day 39 (−0.46). Relative proportions of *E. coli* and Enterobacteria were all significantly reduced one week after weaning (day 6 versus day −1), between days 6 and 19, and again between days 19 and 39 (Table 4).

At weaning, no significant differences in the species richness and alpha diversity (Chao-1 and phylogenetic diversity) were observed in any samples (Table 5). Likewise, no significant differences in the diversity of species or relative abundance of bacterial families (*p* > 0.05) were observed. However, at 6 days post weaning, pigs selected for neomycin treatment (but before treatment commenced) showed reduced species richness and alpha diversity in bacterial taxa and dissimilar microbial diversity relative to all other treatments.

**Figure 3:**
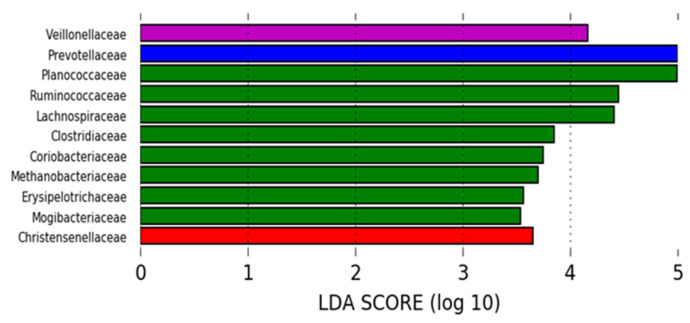
Linear discriminate analysis highlighting the bacterial groups (OTUs) that characterize the microbiomes of pigs selected for each treatment at 6 days post weaning, after two bromelain doses (green), but before the commencement of neomycin sulfate (blue), zinc oxide (purple) or control (red) treatments.

However, at 6 days post weaning, pigs selected for neomycin treatment (but before treatment commenced) showed reduced species richness and alpha diversity in bacterial taxa and dissimilar microbial diversity relative to all other treatments (*p* < 0.05). Neomycin pigs had increased relative abundances of *E. coli* and *Enterobacteriaceae* relative to control and bromelain-treated (2 doses) pigs (Table 6) and an increased abundance of *Prevotellaceae* relative to all other treatments (Figure 3). Pigs selected for zinc treatment only differed by an increased abundance of *Veillonellaceae,* whereas bromelain-treated pigs had an increased relative abundance of *Ruminococcaceae*, *Lachnospiraceae*, *Mogibacteriaceae*, *Clostridiaceae*, *Erysipelotrichaceae*, *Planococcaceae*, *Coriobacteriaceae* and *Methanobacteriaceae*. Control pigs were characterized by an increased abundance of *Christensenellaceae* and a higher proportion of pathogenic F4 *E. coli* relative to total *E. coli* (Figure 3, Table 6, Supplementary Table S1). 

**Figure 4:**
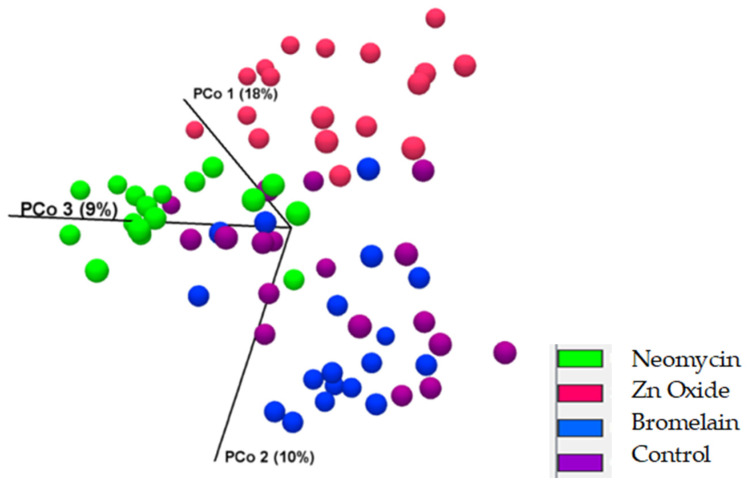
PCA of beta diversity (Bray–Curtis) showing distinct microbial population clustering between treatments at day 19 (13 days after in-feed treatments commenced).

Thirteen days after in-feed treatment commenced (Day 19), significant dissimilarities in the diversity of microbial populations were demonstrated between treatments (*p* = 0.0001 and Figure 4), with pairwise comparisons showing the greatest dissimilarity between control and both neomycin- and zinc-treated pigs (*p* = 0.00002), and significant dissimilarity between bromelain-treated and control pigs (*p* = 0.005). Bacterial diversity was also significantly different between bromelain- and both neomycin- and zinc-treated pigs (*p* = 0.00001) and between zinc- and neomycin-treated pigs (*p* = 0.00001). Zinc-treated pigs showed a reduced phylogenetic diversity in bacterial taxa within samples compared with neomycin-treated pigs (Table 5). 

**Figure 5:**
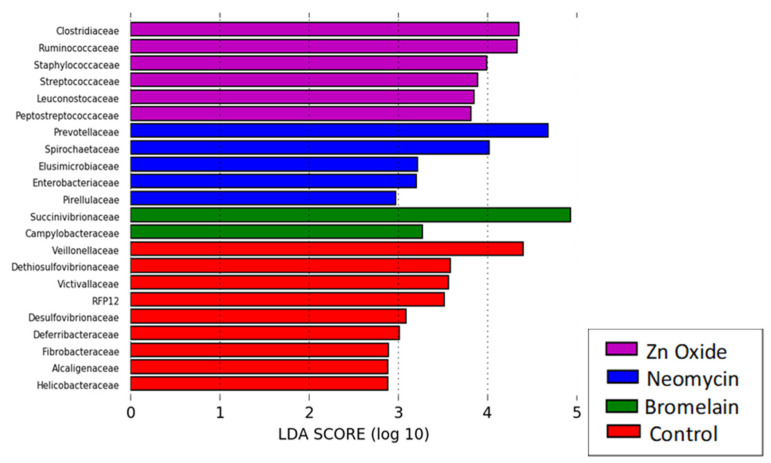
Linear discriminate analysis (LDA) showing the characteristic bacterial groups (OTUs) in each treatment’s fecal microbiome following 13 days of in-feed treatments (day 19 post weaning).

Neomycin-treated pigs were characterized by an increased abundance of *Prevotellaceae*, *Spirochaetaceae*, *Elusimicrobioaceae*, *Pirellulaceae*, *Enterobacteriaceae* (Figure 5) and *Escherichia coli* (Table 6). However, neomycin-treated pigs also maintained the lowest relative abundance of ETEC F4 *E. coli* at day 19. Zinc-treated pigs showed an increased abundance of *Leuconostocaceae*, *Streptococcaceae* and *Staphylococcaceae*, *Ruminococcaceae*, *Clostridiaceae*, and *Peptostreptococcaceae* (Figure 5) and relatively fewer *Bifidobacteria* spp. (*p* = 0.025). Bromelain-treated pigs were characterized by an increased abundance of *Succinivibrionaceae* and *Campylobacteriaceae*, and control pigs showed an increased abundance of *Veillonellaceae*, *Dithiosulfovibrionaceae*, *Victivallaceae*, *RFP12*, *Desulfovibrionaceae*, *Deferribacteriaceae*, *Fibrobacteriaceae*, *Alcaligenaceae* and *Helicobacteriaceae*. 

Disturbances to the fecal microbiome of weaners observed during treatment appeared to resolve 20 days after treatment ceased (day 39). However, significant dissimilarities in the diversity of treatment microbiomes persisted (*p* = 0.0005), with pairwise comparisons showing dissimilarity between control and both neomycin- (*p* = 0.023) and bromelain-treated pigs (*p* = 0.007), but no significant dissimilarity between control and zinc-treated pigs (*p* = 0.096). The bacterial community diversity was also significantly different between bromelain- and both neomycin- (*p* = 0.007) and zinc-treated pigs (*p* = 0.015) and between zinc- and neomycin-treated pigs (*p* = 0.02). There was no significant difference in alpha diversity in bacterial taxa (Chao-1 and phylogenetic diversity) between treatments (Table 5).

**Figure 6:**
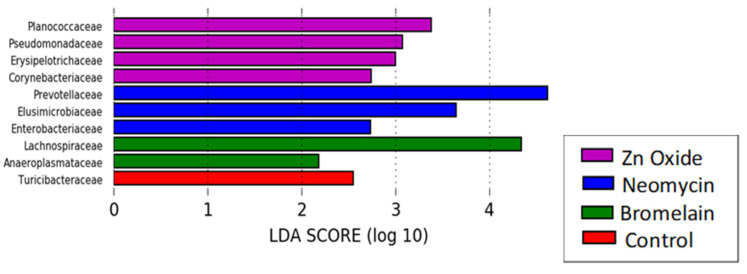
Linear discriminate analysis (LDA) showing the characteristic bacterial groups (OTUs) in each treatment’s fecal microbiome 20 days after in-feed treatment ceased (day 39 post weaning).

Neomycin-treated pigs continued to have a higher abundance of *Prevotellaceae*, *Elusimicrobioaceae* and *Enterobacteriaceae* (Figure 6). Zinc-treated pigs showed an increased relative abundance of *Planococcaceae*, *Pseudomonadaceae*, *Erysipelotrichaceae* and *Corynebacteriaceae*. Bromelain-treated pigs were characterized by an increased abundance of *Lachnospiraceae* and *Anaeroplasmataceae*, and controls had a higher abundance of *Turicibacteraceae*. Control and zinc-treated pigs also had an increased relative proportion of pathogenic F4 ETEC at day 39 (Table 6), after treatment ceased.

### 3.4. D- and L-Lactate Measurements

Neomycin- and zinc-treated pigs showed significantly reduced D-lactate fecal concentrations at day 19 relative to control pigs (Table 7), with similar L-lactate concentrations between treatments at days 19 and 39. Time had no significant effect on D- and L-lactate fecal concentrations (*p* = 0.09). Two weeks after treatments ceased (day 39), both D- and L-lactate fecal concentrations were similar between treatments (*p* > 0.05). 

### 3.5. Antimicrobial Resistance

In the pre-treatment period (day 6), resistance to amoxicillin and tetracycline was fairly high in all *E. coli* isolates (31% and 66%, respectively). There were no differences in the frequency of *E. coli* with individual or multidrug antimicrobial resistance between the assigned treatment groups, except for increased tetracycline resistance in control pigs (*p* = 0.002). No resistance to ceftiofur or neomycin was observed in any *E. coli*, and only low levels of resistance to apramycin (2.7%), lincospectin (13.9%) and sulfamethoxazole/trimethoprim (8.0%) were detected. 

**Figure 7:**
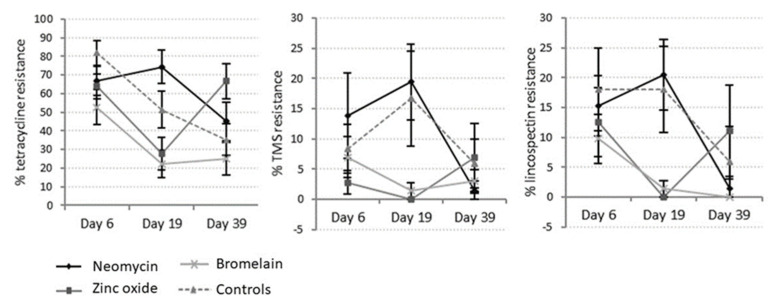
Percentage of *E. coli* isolates with resistance to tetracycline, lincospectin and sulphamethoxazole/trimethoprim (TMS) in pigs treated with neomycin, zinc oxide, bromelain or control pigs.

*E. coli* isolates from day 19 feces continued to show high levels of resistance to amoxicillin (34%) and tetracycline (45.5%), no resistance to ceftiofur and low levels of resistance to apramycin (7.2%), neomycin (3.5%), lincospectin (9.5%) and sulfamethoxazole/trimethoprim (8.9%). Treatment of pigs with neomycin sulfate for 14 days significantly increased *E. coli* resistance to neomycin, apramycin and tetracycline relative to other treatments (*p* ≤ 0.01). In contrast, treatment with bromelain or zinc oxide reduced *E. coli* resistance to lincospectin, tetracycline and sulfamethoxazole/trimethoprim (TMS) relative to controls and neomycin-treated pigs (*p* < 0.001) in the treatment period (Figure 7). The proportion of *E. coli* with multidrug resistance was also lowered in bromelain- and zinc-treated pigs relative to neomycin-treated and control pigs (Table 8) at day 19. Time had no effect on the proportion of *E. coli* with multidrug resistance (repeated-measures ANOVA F probability = 0.153).

Three weeks after all treatments ceased (day 39), a high proportion of *E. coli* isolates continued to show resistance to amoxicillin and tetracycline (45% and 43%, respectively). Only low levels of resistance to apramycin (4.0%), neomycin (2.2%), lincospectin (4.7%) and TMS (4.4%), and no resistance to ceftiofur, were observed. Pigs treated with zinc oxide and neomycin had a higher percentage of *E. coli* with resistance to tetracycline (*p* < 0.001). Increased *E. coli* resistance to lincospectin (*p* = 0.022) and multidrug resistance were also more prevalent in zinc-treated pigs compared with control and bromelain-treated pigs (Table 8). Control pigs expressed a significantly reduced resistance to amoxicillin (*p* = 0.003) relative to all other treatments. 

The sampling time affected amoxicillin resistance, with significantly more *E. coli* expressing resistance after treatment ceased (*p* < 0.001). Conversely, significantly fewer examples of tetracycline-resistant *E. coli* were identified during and after treatment (*p* = 0.003) and fewer lincospectin-resistant *E. coli* (*p* = 0.04) were detected after treatment relative to other periods. 

## 4. Discussion

Neomycin sulfate, zinc oxide and bromelain all helped control post-weaning diarrhea (PWD) in this study. Fecal consistency, the duration of diarrhea and the number of electrolyte doses were not significantly affected by treatment during the two-week treatment period, but diarrhea severity did increase in pigs previously treated with zinc once treatment ceased. Only neomycin treatment increased weight gain and feed intake in pigs in the five weeks post weaning. 

Pigs were affected by a low level of diarrhea and low numbers of F4 ETEC excreted in the first two weeks post weaning. The high health status of the herd and the provision of a probiotic *Lactobacillus* species specific to the source farm pre-weaning may explain the low numbers of pathogenic F4 ETEC detected. In addition, other risk factors for PWD such as fluctuating temperatures, draughts, poor hygiene, early weaning and high-protein diets [27,28,29] were not a feature in this trial. Although about 40% of the trial pigs carried receptors making them genetically susceptible to F4 ETEC [30,31], the presence of the MUC4 receptor had no effect on the relative abundance of F4 ETEC in mucosal scrapings or feces. Comingling and random allocation of piglets at weaning did not affect relative numbers of excreted F4 *E. coli* nor fecal microbial communities between treatments before the trial commenced. 

Previous studies have demonstrated that zinc oxide [27,32], neomycin sulfate [15] and bromelain [20,33] can reduce diarrhea and improve growth in weaners naturally or experimentally challenged with F4 ETEC, but these studies involved either more genetically susceptible pigs, higher doses of F4 ETEC, higher dietary protein or poor environmental and hygiene conditions. The reported efficacy of zinc to either reduce diarrhea or the number of ETEC also depends on the duration of the study, with antibacterial properties only evident in the first week post weaning [34,35]. 

Neomycin treatment reduced the relative abundance of pathogenic F4 ETEC to total *E. coli* shed in feces during the 14-day treatment period, which persisted for an additional three weeks after treatments ceased. Neomycin-treated pigs also showed an increased abundance of non-F4 *E. coli* and *Enterobacteriaceae* in the absence of diarrhea, suggesting that commensal *E. coli* may have colonized the GIT. Commensal *E. coli* can competitively exclude pathogenic bacteria using bacteriocins and antibacterial peptides [36]. 

In our study, pharmacological doses of dietary zinc fed to weaners did not lead to a reduced relative abundance of F4 ETEC, *E. coli* or *Enterobacteriaceae,* in agreement with previous reports [23,37]. Instead, zinc oxide is reported to reduce the incidence of diarrhea by inhibiting adhesion and internalization of ETEC into the intestinal epithelium, thus preventing disruption to the expression of tight junction proteins and protecting intestinal integrity [19]. Increased intestinal integrity was demonstrated in both the zinc- and neomycin-treated pigs in our trial, shown by the reduced D-lactate levels in their feces and in digesta, indicating that D-lactate produced by lactic acid bacteria was not able to translocate across the epithelium as previously described in animals with increased intestinal permeability [38,39]. Increased intestinal integrity in neomycin-treated pigs was also demonstrated by the increased abundance of the bacterial family *Pirellulaceae*, reported to be correlated with increased expression of tight junction proteins between cells [40]. Through increased intestinal integrity, the epithelium of zinc- and neomycin-treated pigs was protected from F4 ETEC colonization and translocation of enterotoxins [41]. However, this protection was transient, as the severity of diarrhea and the relative abundance of F4 ETEC increased after zinc treatment ceased. 

Bromelain treatment reduced the proportion of pathogenic F4 ETEC shed in feces, along with increasing the proportion of total *E. coli*. Bromelain has no bactericidal activity; instead, it acts by specifically cleaving F4 ETEC receptors on host enterocytes, preventing the attachment and injection of F4 ETEC bacteria and toxins to the pig intestinal epithelium [21,22]. Bromelain treatment did not exert any selection pressure on intestinal *E. coli* to develop resistance to antibiotics commonly used in weaner pigs.

While all treatments have previously demonstrated efficacy in controlling PWD, this is the first report outlining the impact of PWD treatments on the intestinal microbiome of weaned pigs. The intestinal microbiomes of pigs treated with bromelain remained relatively similar to those of control pigs, with an increased abundance of commensal bacterial families relative to zinc- and neomycin-treated pigs. Bromelain-treated pigs avoided intestinal dysbiosis and the loss of commensal bacteria commonly observed in the first two weeks post weaning and avoided the loss in species diversity observed in zinc-treated pigs. Specifically, bromelain-treated pigs showed an increased relative abundance of butyrate-producing bacteria reported to ferment undigested complex oligosaccharides and polysaccharides into butyrate in the colon [42]. Initially, the microbiomes of control pigs were characterized by an increased abundance of the butyrate -producer *Christensenellaceae*. Previous studies demonstrated that improved control of PWD is associated with increased abundance of butyrate-producing bacterial families including *Ruminococcaceae, Lachnospiraceae*, *Christensenellaceae* and *Erysipelotrichaceae* [24].

Commensal bacteria in the intestine play an important role in preventing the colonization of pathogens through competitive exclusion and the excretion of bacteriocins capable of bacterial lysis [5]. In addition, bacterial production of butyrate in the colon can reduce inflammation by promoting the differentiation of T regulatory cells from pro- to anti-inflammatory cytokines and can improve intestinal function through increased villous height and increased intestinal barrier function by inducing genes encoding tight junctions between epithelial cells [9].

Diarrhea in control pigs, between 5 and 8 weeks of age, led to intestinal dysbiosis. Affected controls showed a reduced abundance of commensal bacteria and increased *Veillonellaceae* and *Alcaligenaceae* abundance, reported previously in pigs affected by PWD [24,43]. Bromelain-treated pigs were characterized by an increased relative abundance of *Succinivibrionaceae*, a family of bacteria able to ferment undigested carbohydrates to form acetate and succinate, providing the host with fuel for oxidative phosphorylation via the citric acid cycle [44]. However, an increased abundance of *Succinivibrionaceae* has also been reported to be associated with increased diarrhoea incidence [34], low residual feed intake [45,46] and reduced weight gain [47]. Only reduced feed intake was observed in association with increased *Succinivibrionaceae* in this study.

Treatment with neomycin sulfate induced significant changes in the intestinal microbiome (dysbiosis) in the first two weeks post weaning. Neomycin-treated pigs were characterized by an increased abundance of *Enterobacteriaceae* and *Proteobacteria* and a reduced abundance of commensals, specifically lactate- and butyrate-producing bacteria. Elevated *Proteobacteria* are reported to be markers of dysbiosis or unstable intestinal microbiomes and colitis in mammals [48]. Broad-spectrum antibiotics like neomycin are bactericidal against both F4 ETEC and commensals in the GIT, inducing dysbiosis and reduced diversity of the fecal microbiome, as reported previously [49,50,51,52,53,54,55]. 

Mortalities in neomycin-treated pigs caused by *Streptococcus suis* infection and chronic pericarditis may be related to dysbiosis or damage to the protective immune function of the GIT. Intestinal dysbiosis has been reported to aggravate lung injury associated with *S. suis* infection by affecting alveolar macrophage activity and the Th1/Th2 balance [56]. 

Neomycin treatment also exerted selection pressure on intestinal *E. coli* to develop resistance to aminoglycosides (neomycin and apramycin), as well as other antibiotics commonly used to treat PWD including tetracycline, lincospectin and TMS. Disturbingly, oral administration of aminoglycosides to pigs carries a very high risk of increasing the prevalence of antimicrobial resistance in *E. coli* [57]. While *E. coli* resistance to aminoglycosides appeared to be short-lived, resistance to tetracycline persisted for at least three weeks after treatment ceased. 

However, not all microbiome changes in neomycin-treated pigs were detrimental. Increased abundance of *Prevotellaceae* was observed throughout the trial and is often associated with weaning and increased feed intake [49,58,59]. *Prevotellaceae* are required in increasing abundance to ferment plant-derived non-starch polysaccharides to short-chain fatty acids in the colon [60] associated with the post-weaning change from a milk-based to a carbohydrate diet.

The microbiomes of pigs fed pharmacological doses of zinc oxide led to reduced bacterial richness (Chao-1) and dysbiosis, in agreement with previous reports, in the colon and cecal contents of PWD-affected weaners [61,62]. Zinc treatment led to an increased abundance of some commensals including *Clostridiaceae*, *Ruminococcaceae* and *Leuconostocaceae* but also a reduced relative abundance of *Bifidobacteria* spp. and an increased abundance of families associated with increased GIT inflammation like *Streptococcaceae*, as reported previously [34,63,64]. Dysbiosis persisted for at least three weeks after zinc treatment ceased, as demonstrated previously [65], due to the bacteriostatic effect of zinc on a wide range of intestinal microbiota. Zinc treatment initially appeared to reduce *E. coli* resistance to aminoglycosides, tetracycline, lincospectin and TMS, but once zinc was removed from pig diets, the prevalence of *E. coli* resistance to TMS, lincospectin and tetracycline increased. Multidrug-resistant *E. coli* were also more prevalent three weeks after zinc treatment ceased, in agreement with earlier studies [66].

## 5. Conclusions

By preventing F4 ETEC attachment to receptors on intestinal enterocytes, bromelain treatment pre-weaning prevented diarrhea and production losses, but importantly, bromelain had no antimicrobial effects against any other bacteria. Consequently, the commensal microflora of bromelain-treated pigs remained stable, and their ability to produce short-chain fatty acids for colonocytes and to competitively exclude pathogen attachment and invasion remained functional. Microbial diversity in the weaner pig intestine is dynamic, increasing in richness due to the diversity of substrates [60] and reducing in diversity because of disease and antimicrobial treatments. In this study, we observed a disruption to the intestinal microbiome in response to diarrhea and to treatment with both neomycin and zinc oxide. 

Additional advantages of bromelain treatment include the absence of any selection pressure on bacteria to develop resistance to antimicrobials. In contrast, single-drug and multidrug antibiotic resistance was increased in commensal *E. coli* treated with neomycin or zinc, potentially reducing the longevity of critical antibiotics in human disease outbreaks and nosocomial infections. Pig farms worldwide already endeavour to reduce risk factors associated with PWD including feeding weaner diets with reduced dietary protein, improving hygiene, reducing temperature fluctuations and draughts and breeding pigs without enterocyte receptors for attachment of ETEC. Including pre-weaning treatment of weaners with bromelain into routine weaner management will reduce the reliance on antibiotics and heavy metals for disease control, which will ultimately benefit both pig and human health.

## Figures and Tables

**Table 1:** Clinical signs and electrolyte treatments of pigs before treatment (days 0 to 6), during treatment (days 7 to 20) and after treatment (days 21 to 39).

	Number of Pigs with Diarrhea Score ≥ 2				
Days	Neomycin	Zinc Oxide	Bromelain	Control	*p* Value
0 to 6	0	3	0	2	0.14
7 to 13	0	2	5	3	0.20
14 to 20	1	1	1	5	0.22
21 to 39	6	6	2	2	0.38
	Mean number of days of electrolyte treatment				
0 to 6	0	4	0	0	0.13
7 to 13	0	4	2	3	0.38
14 to 20	0	4	0	3	0.32
21 to 39	6	5	4	2	0.61

**Table 2:** Percent water content in fecal samples of pigs before treatment (days 0 to 6), during treatment (days 7 to 20), after treatment (days 21 to 39) and in digesta at necropsy (day 40).

	Day 6	Day 19	Day 39	Digesta
Neomycin	68.61	73.77	72.30 ^a^	82.16 ^a^
Zinc oxide	69.65	74.83	73.64 ^b^	82.33 ^a^
Bromelain	68.23	73.41	72.11 ^a^	79.64 ^b^
Control	67.55	75.19	73.11 ^ab^	82.47 ^a^

Different superscripts within columns indicate significant differences.

**Table 3:** Predicted weight gain, feed intake and mortalities blocked by replicate, gender, litter and interactions with starting weight as a covariate (unbalanced ANOVA).

Treatment	Weaning Weight (kg)	Weight Gain Day 7 to 39 (kg)	Feed Intake Day 7 to 39 (kg)	Feed to Gain Day 7 to 39 (kg)
Neomycin	6.30	23.23 ^a^	25.77 ^ad^	1.116
Zinc oxide	6.30	22.02 ^ab^	24.45 ^ac^	1.112
Bromelain	6.32	21.07 ^b^	23.66 ^bc^	1.133
Control	6.54	22.77 ^ab^	27.00 ^d^	1.183
*p* value ^1^	0.517	0.038	<0.001	

^1^ Different superscripts within columns indicate significant differences.

**Table 4:** Change in mean relative ratios of bacteria (log_10_-transformed) in pig feces from all treatments after weaning (days −1 to 6), during treatment (days 7 to 19) and after treatment (days 19 to 39).

Treatment	Day −1	Day 6	Day 19	Day 39
F4 ETEC: *E. coli*	−3.60 ^a^	−2.19 ^b^	−1.65 ^c^	−0.46 ^d^
*E. coli*: total bacteria	−1.09 ^a^	−3.63 ^b^	−4.09 ^c^	−4.82 ^d^
Enterobacteria: total bacteria	−1.17 ^a^	−3.78 ^b^	−4.30 ^c^	−5.02 ^d^

^abcd^ Different superscripts within rows indicate significant differences.

**Table 5:** Species richness and alpha diversity (Chao-1 and phylogenetic diversity) of microbial communities within treatments at days −1, 6, 19 and 39.

	Chao-1 Bias-Corrected				Phylogenetic Diversity			
Treatment	Day −1	Day 6	Day 19	Day 39	Day −1	Day 6	Day 19	Day 39
Neomycin	579	538 ^a^	615	564	2.65	2.68 ^a^	2.91 ^a^	2.88
Zinc oxide	572	690 ^b^	591	563	2.61	3.00 ^b^	2.61 ^b^	2.85
Bromelain	559	719 ^b^	622	600	2.49	2.93 ^ab^	2.87 ^ab^	2.86
Control	545	758 ^b^	600	591	2.54	2.96 ^ab^	2.71 ^ab^	2.78
*p* value ^1^	0.505	<0.0001	0.673	0.418	0.360	0.01	0.007	0.759

^1^ Different superscripts within rows indicate significant differences.

**Table 6:** Relative ratios of bacterial groups (log_10_-transformed) in pig feces before treatment (day 6), during treatment (day 19) and after treatment (day 39).

Bacterial Ratios (log_10_)	Neomycin	Zinc Oxide	Bromelain	Control	*p* Value
	Day 6 (Before Treatment)				
F4 ETEC: total *E. coli*	−2.613 ^a^	−2.077 ^ab^	−2.211 ^ab^	−1.809 ^b^	0.042
*E. coli*: total bacteria	−3.212 ^a^	−3.588 ^ab^	−3.572 ^bc^	−4.061 ^c^	0.006
Enterobacteria: total bacteria	−3.259 ^a^	−3.662 ^ab^	−4.077 ^b^	−4.186 ^b^	0.011
	Day 19 (During treatment)				
F4 ETEC: total *E. coli*	−2.292 ^a^	−1.526 ^b^	−1.402 ^b^	−1.385 ^b^	0.025
*E. coli*: total bacteria	−3.491 ^a^	−4.091 ^ab^	−4.466 ^b^	−4.271 b	0.040
Enterobacteria: total bacteria	−3.687	−4.351	−4.761	−4.400	0.085
	Day 39 (After treatment)				
F4 ETEC: total *E. coli*	−1.461 ^a^	0.164 ^b^	−0.907 ^a^	0.430 ^b^	<0.001
*E. coli*: total bacteria	−3.855 ^a^	−5.405 ^b^	−4.367 ^a^	−5.654 ^b^	<0.001
Enterobacteria: total bacteria	−4.130 ^a^	−5.228 ^bc^	−4.703 ^ab^	−6.040 ^c^	0.005

Different superscripts within rows indicate significant differences.

**Table 7:** Concentrations of D- and L-lactate in pig feces during treatment (day 19) and after treatment ceased (day 39).

	D-Lactate (mM) in Dry Matter		L-Lactate (mM) in Dry Matter	
Treatment	Day 19	Day 39	Day 19	Day 39
Neomycin	1.618 ^a^	1.503	1.189	1.127
Zinc oxide	2.032 ^a^	2.619	1.329	1.297
Bromelain	2.433 ^ab^	1.769	1.705	1.461
Control	2.993 ^b^	2.916	1.846	2.166
*p* value ^1^	0.027	0.139	0.142	0.203

^1^ Different superscripts within columns indicate significant differences.

**Table 8:** The percentage of multidrug-resistant *E. coli* from pig feces before treatment (day 6), during treatment (day 19) and after treatment ceased (day 39).

Treatment	Day 6	Day 19	Day 39
Neomycin	35 ^a^	36 ^ab^	17 ^ab^
Zinc oxide	42 ^a^	18 ^b^	37 ^b^
Bromelain	19 ^b^	19 ^b^	9 ^a^
Control	30 ^a^	42 ^a^	15 ^a^

Different superscripts within columns indicate significant differences.

## Data Availability

The data presented in this study are available on request from the corresponding author. The data are not publicly available due to privacy restrictions placed by the funding body CRC HIAP.

## Supplementary Materials

The following supporting information can be downloaded at: https://www.mdpi.com/article/10.3390/ani13203229/s1, Table S1: Relative abundance of bacterial families in pig feces before treatment (day 6), during treatment (days 19) and after treatment (day 39).
