# Supplementary material for: Pre-Treatment with Bromelain Prevents Intestinal Dysbiosis in Pigs with Post-Weaning Diarrhea, without Increasing Antimicrobial Resistance in Escherichia coli
Source: Animals (Basel). 2023 Oct 16;13(20):3229. doi: 10.3390/ani13203229 (PMC10603644; doi:10.3390/ani13203229)
Supplement: Supplementary file 1 [file animals-13-03229-s001.zip › animals-2543558-supplementary.pdf]

Supplementary Table S1. Relative abundance of bacterial families in pig feces before treatment (day 6), during treatment (days 19) and after treatment (day 39).

			**Before treatment (day 6)**		
**Bacterial Family**	**Neomycin**	**Zinc oxide**	**Bromelain**	**Control**	**P value**
Erysipelotrichaceae	0.63^{a}	0.96^{b}	1.20^{b}	1.19^{b}	0.032
Lachnospiraceae	6.60^{a}	9.90^{bc}	11.36^{b}	8.72^{ac}	0.006
Ruminococcaceae	7.26^{a}	10.07^{b}	11.67^{b}	11.77^{b}	0.002
Prevotellaceae	56.06^{a}	41.16^{b}	35.34^{c}	39.13^{bc}	<0.001
Succinivibrionaceae	1.90^{a}	0.75^{b}	0.51^{b}	0.82^{b}	0.010
Veillonellaceae	9.53^{a}	12.02^{b}	11.31^{ab}	12.12^{b}	0.024
Clostridiaceae	1.46^{a}	1.68^{a}	3.03^{b}	2.14^{a}	0.001
			During treatment (Day 19)		
Desulfovibrionaceae	0.083^{a}	0.027^{b}	0.094^{a}	0.130^{a}	0.003
Streptococcaceae	0.49^{a}	1.75^{b}	0.30^{a}	0.26^{a}	0.007
Erysipelotrichaceae	0.55^{a}	0.88^{b}	0.60^{a}	0.76^{ab}	0.013
Ruminococcaceae	6.22^{a}	8.69^{b}	4.70^{c}	6.10^{ac}	<0.001
Prevotellaceae	47.38^{a}	44.09^{b}	38.30^{c}	42.07^{b}	<0.001
Succinivibrionaceae	1.97^{a}	0.89^{a}	16.75^{b}	9.03^{c}	<0.001
Clostridiaceae	2.52^{a}	5.87^{b}	1.82^{a}	1.61^{a}	<0.001
			After treatment (Day 39)		
Erysipelotrichaceae	0.26^{a}	0.44^{b}	0.38^{b}	0.42^{b}	0.015
Lachnospiraceae	7.92^{a}	8.08^{a}	12.17^{b}	9.11^{a}	<0.001
Prevotellaceae	39.36^{a}	37.31^{a}	35.02^{ab}	31.59^{b}	0.005
Veillonellaceae	11.84^{a}	15.28^{b}	11.90^{a}	15.13^{b}	0.002
Succinivibrionaceae	10.95^{a}	11.29^{a}	11.44^{a}	17.72^{b}	0.004
Enterobacteriaceae	0.034^{a}	0.008^{b}	0.008^{b}	0.003^{b}	<0.001
Lactobacillaceae	0.64^{a}	1.38^{b}	0.91^{ab}	0.77^{a}	0.046
